# Study on the influence of overlying coal seam mining on the deformation of floor roadway surrounding rock

**DOI:** 10.1038/s41598-025-22159-z

**Published:** 2025-11-03

**Authors:** Zhongsi Dou, Shixiang Xu, Ruili Han

**Affiliations:** 1 School of Civil and Architectural Engineering, East China University of Technology, Nanchang, 330013 China; 2 Engineering Research Center for Digital Risk Control of Underground Engineering of Jiangxi Province, East China University of Technology, Nanchang, 330013 China; 3 School of Earth Sciences, East China University of Technology, Nanchang, 330013 China

**Keywords:** In-situ stress, Mining-induced stress, Physical modeling experiment, Coal mine roadway, Deformation damage, Energy science and technology, Engineering, Environmental sciences, Natural hazards, Solid Earth sciences

## Abstract

The roadway is the lifeline of the underground production system of the coal mine. Once deformation and damage occur, it can easily lead to mining disasters. After the coal seam is mined, the distribution of the original stress field is changed, and a new mining stress field is generated, which causes the surrounding rock of the roadway at the bottom of the coal seam to be affected to varying degrees. This paper takes the Lu Ling coal mine in China as the research object, and uses the method of combining similarity simulation test and numerical simulation to study the surrounding stress and deformation damage of the floor roadway with the mining of the main coal seam in Luling Coal Mine. The results show that under the action of tectonic stress, with the mining of the overlying main coal seam, the stress concentration and plastic damage of the roof and floor of the roadway in the coal seam floor are relatively small, while the pressure relief and plastic damage of the two sides are relatively large in Luling Coal Mine. To verify the influence of different mining dynamic stresses on the deformation and damage of roadway, mining simulations were conducted under both structural stress and self weight stress conditions. The results showed that the collapse shape of the roof was significantly different under different stress fields, and the stress evolution law of the mining floor was also different. Under the main control of structural stress, the total body of the floor was mainly under compressive stress, while under the main control of self weight stress, it was significantly affected by the unloading of the overlying goaf, mainly under tensile stress, resulting in significantly different forms of roadway failure. This study can provide a reference for analyzing the surrounding rock stability of all-mudstone roadways in mines under different in-situ stress conditions.

## Introduction

With the exploitation of coal resources extending into deeper strata, the stability issues of soft rock roadways under high in-situ stress environments have become increasingly prominent^1^. In-situ stress is the fundamental driving force behind the deformation and failure of roadway surrounding rock. Roadway excavation disrupts the original in-situ stress field, inducing stress redistribution within a specific zone. The stability of the surrounding rock is directly dependent on whether its strength can withstand the redistributed stress field^2–4^.The in-situ stress field, as the core representation of the original mechanical state of rock masses, exerts a decisive influence on the deformation and failure mechanisms of roadway surrounding rock through its heterogeneity and dynamic evolution characteristics^5^.Therefore, studying the law of deformation and failure of roadway surrounding rock caused by mining-induced stress evolved from in-situ stress has important significance^6^.

Scholars have analyzed and summarized the macro-laws of the in-situ stress field based on actual in-situ stress measurement data, and obtained a large number of valuable research results. Using in-situ stress measurement data, Hast^7^ studied the stress field in the Scandinavian region of Finland. Through regression analysis, it was found that the magnitude of horizontal stress increases linearly with depth. Zoback et al.^8^ plotted the world in-situ stress map by analyzing in-situ stress data from various countries around the world.Since the mid-20th century, with the refinement of the development direction of rock mechanics, significant progress has been made in the study of the stress, deformation and failure mechanisms of various roadways (such as coal roadways and rock roadways) in mines.The research methods have evolved from the original elastic and elastoplastic approaches to rheology, damage, dilatancy, fracture, etc. With the rapid development of computer science and technology, some auxiliary software has emerged, providing a strong and effective guarantee for revealing the deformation mechanism of roadways^9–12^.

Coggan et al.^13^. used different numerical modeling techniques, such as continuum, discontinuum, and finite element-discrete element hybrid algorithms, to model the deformation characteristics of coal measure strata. Combined with specific case studies, they discussed the application and applicability of these techniques in modeling weak rock masses. The simulation results show that the thickness of relatively weak mudstone in the tunnel roof has a significant impact on the failure range and ultimately determines whether additional support measures are required. Zhao et al.^14^ conducted a systematic study on the stress characteristics, failure mechanisms, and control methods of the gob-side roadway (GSR) in the Hong qinghe Coal Mine of the Xinjie deep mining area, Inner Mongolia, China. By establishing long-short arm F-shaped roof structure models for different mining stages of the first working face, they analyzed the deformation and failure laws of the GSR surrounding rock based on the superposition effect of three loads. Sun et al.^15^ carried out research on the severe deformation and failure problems of mining roadways under high secondary stress conditions in Xuchang Coal Mine, Shandong Province. Through field measurements and numerical simulation analyses, it was found that the superposition effects of advance abutment pressure, lateral abutment pressure, and fault structures (stress concentration factor K = 2.3) significantly influence the distribution of the maximum principal stress in the surrounding rock of the roadways. Tian et al.^16^ analyzed the characteristics and types of non-uniform pressure stress fields through detailed numerical simulations. Wu et al.^17^ studied the effects of different in-situ stress fields and dynamic loading conditions on surrounding rock through numerical simulations. They found that horizontal stress, vertical stress, and mining-induced stress are positively correlated with the plastic failure of the surrounding rock, and the influence of horizontal stress on roadway deformation is more significant than that of vertical stress. Meng et al.^18^ conducted a study on roadway deformation and failure by combining numerical simulation and field observation. They expounded the deformation mechanism of deep soft-rock roadways under the action of mining-induced stress and proposed corresponding control measures. Wang et al.^19^ established a three-dimensional numerical model of surrounding rock damage under load using FLAC3D software to study the laws of displacement, stress, and plastic expansion during the damage and failure evolution of roadway surrounding rock. Through MATLAB numerical analysis, they verified the mechanical response mechanisms of bending deformation, elastoplastic transformation, and unloading failure of the surrounding rock in deep soft rock roadways, and revealed the spatio-temporal evolution characteristics of soft rock deformation and failure. Tian et al.^16^. analyzed the characteristics and types of non-uniform pressure stress fields through numerical simulations. The study shows that under mining disturbances, the non-uniform pressure stress fields above roadways mainly exist in two types: the “V-shaped” and “oblique line-shaped” fields. The scope of the roadway plastic zone, the amount of asymmetric deformation, and the non-uniformity index increase with the increase of the off-load coefficient. The failure of the surrounding rock is dominated by shear failure, accompanied by a small amount of local tensile failure. Kong et al.^20^ systematically analyzed the evolution laws of surrounding rock stress fields and displacement fields through physical similarity model tests, numerical simulations, and theoretical analyses. The research shows that the shallow surrounding rock undergoes tensile failure, while the deep surrounding rock experiences shear failure, indicating that different in-situ stress conditions have significant differential effects on the deformation of roadway surrounding rock. Sun et al.^21^. carried out physical modeling experiments to study the deformation mechanism of roadway excavation in deeply buried soft rock strata. Strain rosettes were arranged in a circular pattern within the surrounding rock to monitor its strain. By comprehensively analyzing infrared images, experimental photographs, and displacement field images, the deformation and failure mechanism of the surrounding rock was revealed. Additionally, a numerical model was used to compare the displacement results with those of the physical model.Xu et al.^22^. systematically studied the failure mechanism of surrounding rock in roadways by combining on-site measurements, theoretical analysis, numerical simulation, and industrial tests. Through FLAC3D simulation, they revealed that the stress concentration coefficient, displacement, and height of the plastic zone have a significant positive correlation with the span of the open-off cut. Hu et al.^23^. established a mechanical model of the surrounding rock in the floor roadway by integrating the law of floor stress distribution during the working face mining process. Through comparative verification between analytical calculations and numerical simulations, they obtained the failure mechanism of the roadway surrounding rock and the development law of the plastic zone morphology, but no physical similar simulation was conducted for further verification.Zhang et al.^24^. analyzed the failure characteristics of surrounding rock and the fracture evolution process under different mining stresses by means of physical similar simulation. It was concluded that under the effect of stress concentration, shear failure first occurs at the side of the roadway, and fractures extend from the roadway’s bottom corner to the unsupported area and the low-strength support area.Hu et al.^25^. found through similar simulation tests that both the conventional mechanical properties and long-term creep characteristics of the new material meet the test requirements, which provides strong support for achieving ideal results in similar model tests.Aiming at the problems of severe deformation of the roadway surrounding rock and strong mine pressure behavior, Cao et al.^26^. studied the deformation mechanism of the roadway surrounding rock by combining theoretical analysis, laboratory tests, numerical simulation, and on-site testing, and proposed corresponding surrounding rock control technologies.

To sum up, the existing research has not yet formed a multi-stress field comparison paradigm of “based on the same model and controlling unified variables”, and thus cannot accurately reveal the control mechanism of stress field types on the deformation and failure of floor roadways—which is exactly the core research gap that this study intends to fill. This study innovatively constructs two identical similar material models, applies vertical self-weight stress and horizontal tectonic stress respectively, and combines numerical simulation verification to realize comparative analysis under"a single variable (stress field type)”, providing more reliable methodological support for the stability research of full-mudstone floor roadways.

## Engineering background

**Fig. 1:**
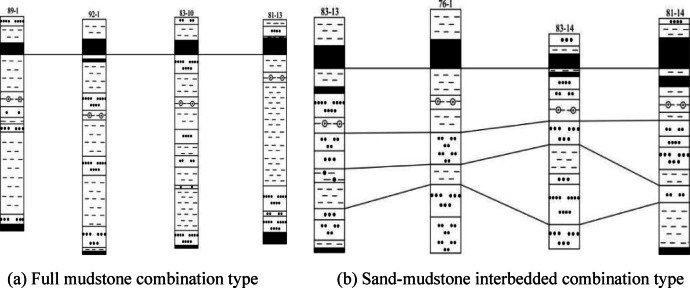
Lithological combination types of the floor of No. 8 coal seam in Luling Coal Mine.

The Lu ling Coal Mine is located in Huaibei, China, and mainly mines the No. 8 coal seam. The average burial depth of this seam is 590 m, and the average thickness is 10.58 m. The floor strata of the No. 8 coal seam and the roadway 35 m below the floor are the research objects of this study. According to the statistical analysis of 75 borehole data from the Luling Coal Mine, it can be concluded that there are mainly two types of lithological combinations developed in the floor of No. 8 coal seam. One is dominated by mudstone with interbedded thin-layer sandstone or entirely composed of mudstone (full mudstone combination type), and the other is dominated by sandstone with interbedded mudstone or sand-mudstone interbedded (sand-mudstone interbedded combination type). The comparison of their stratigraphic columns is shown in Fig. 1.

**Fig. 2:**
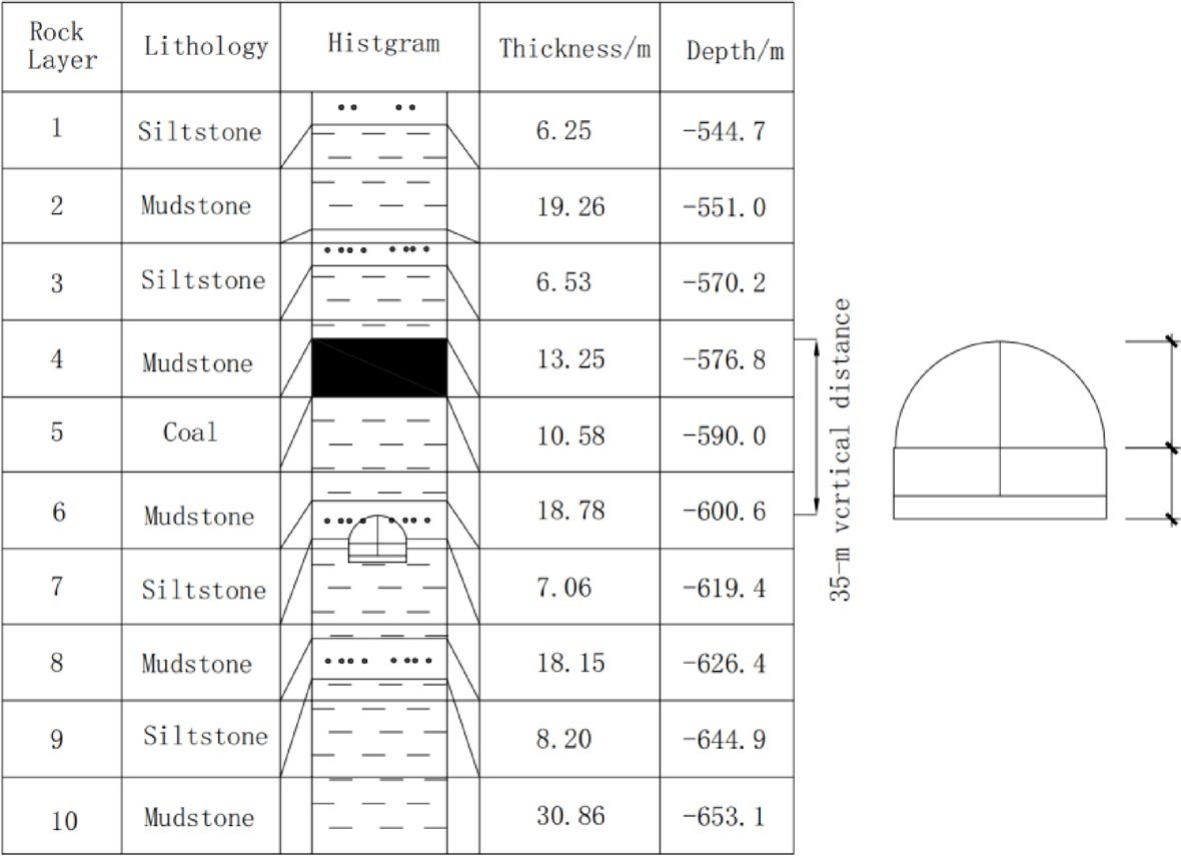
Borehole histogram and floor roadway location map of the mine.

The vertical distance from the floor roadway to the No. 8 coal seam is 35 m. The floor roadway is a soft-rock roadway, and its surrounding rocks are mainly composed of siltstone and mudstone. The height of the vertical wall is 2 m, and the radius of the upper circular arch is 3 m. Figure 2 shows the borehole histogram of the mine.

**Fig. 3:**
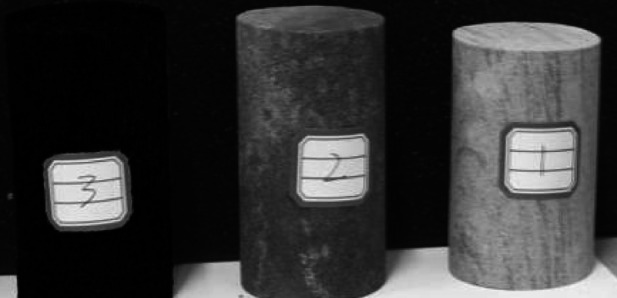
In-stiu sample.

The in-situ rock samples taken from the field are shown in Fig. 3 below. Among them, labels 1, 2, and 3 represent siltstone, mudstone, and raw coal respectively.

The corresponding rock mechanical parameters were obtained through MTS uniaxial compression tests, as shown in Table 1 below.

**Table 1:** Strength test results of rock samples from test Points.

Lithology	Elastic modulus/GPa	Tensile strength/GPa	Compre ssive strength/MPa	Shear strength/MPa
Siltstone	8.06	3.15	18.15	7.56
Mudstone	6.19	2.43	12.36	3.84
raw coal	3.56	1.12	5.78	1.47

In - situ tests on the in - situ stress of mines in the study area were carried out by means of the stress relief method and the AE method. Then, the boundary load stress adjustment method was used to invert the in - situ stress field of the II level (-590 m level) in Luling Mine. As a result, the distribution trend of the in - situ stress field at the second level in the mining area was obtained. The maximum principal stress ranges between 22 MPa and 35 MPa, while the minimum horizontal principal stress ranges between 10 MPa and 16 MPa. Both values are greater than those of the self-weight stress field, indicating that the in-situ stress field in the study area is dominated by the tectonic stress field.

Combined with the engineering practice of the study area, this study selects the full mudstone type of the 8th coal seam floor rock stratum structure in Luling Coal Mine as the simulation object to study the law of mining stress transfer in the coal seam floor and deformation and failure of roadway surrounding rock under the control of horizontal tectonic stress with the mining of overlying coal seams. In order to verify the influence of overlying coal seam mining on the change of floor mining stress under the control of different stress fields, this study innovatively uses the same similar material model to carry out similar material simulation and numerical simulation under the conditions of self-weight stress and tectonic stress respectively.

## Physical modeling experiment

### Selection of similar materials

#### Analog proportion design

The simulation experiment was based on the three theorems of analogy. The ratio of similitude in the simulation was determined as follows.

1. Geometry ratio of similitude: The height of the roadway vertical wall was 2 m, and the radius was 3 m. To avoid size effect, the length × height × thickness of the boundary was 90 m × 60 m × 15 m, and the size of the model was 1.8 m × 1.2 m × 0.3 m. The vertical wall of the simulation roadway was 4 cm with a radius of 6 cm. The geometry ratio is C_L_ =1:45 .
2. Appearance density ratio of similitude: The test on the physical and mechanical parameters of the prototype and standard specimen model indicated that the volume weight ratio of similitude is Cγ = 1:1.2 .
3. Stress ratio of similitude: Cσ = Cγ × C_L_=1:54.
4. Relation between loading of jack and boundary loads: Horizontal loading: the lateral area was 120 cm × 30 cm = 3600 cm^2^. Each lateral area had two pistons. The entire area was 56 cm^2^. Thus, the ratio of lateral oil pressure and lateral boundary loads was 64.286:1; Vertical loading: The roof area was 180 cm × 30 cm = 5400 cm^2^. Two pistons were in the roof. The entire area was 56cm^2^. Thus, the ratio of roof oil pressure and boundary loads was 96.43:1.

#### Matching similar materials and mechanical properties of the test

The proportion of similar materials in this mechanical test was 8:1:0.8, 10:1:1, 12:1:1.2, 14:1:1.4, and 14:1:1.6, according to the ratio of aggregate (sand): cement materials (lime, cement): water. Among them, the proportion between sand and water was fixed at 10:1.Similar materials were placed inside standard cylindrical specimen vessels according to their particular proportion. The materials were divided into several subsections and compactly pressed until the specimen height reached above 100 mm. The produced standard specimens were properly labeled and categorically placed in the laboratory. After approximately 21 d, the specimens were grounded and processed, and their intensity parameters were tested in MTS mechanical testing machines, as shown in Table 2.

**Table 2:** Mechanical parameters of analog simulation material.

Mechanical parameters of rock samples	Similar material ratio, Aggregate: Cement: Water				
	8:1:0.8	10:1:1	12:1:1.2	14:1:1.4	16:1:1.6
Elastic modulus//GPa	0.675	0.501	0.303	0.212	0.151
Compressive strength/MPa	1.029	0.613	0.404	0.232	0.127
Shear strength/MPa	0.398	0.248	0.164	0.130	0.110
Tensile strength/MPa	0.205	0.126	0.108	0.085	0.066

The three experiments revealed that with the decrease of the cement content, the compressive and shearing strengths of the specimens also decreased, whereas the proportion between aggregate and cement was always within a certain scope. On the basis of the strengths of the in situ rocks in the first section, the strengths of siltstone, mudstone, and raw coal were 18, 12, and 6 MPa, respectively. The strengths of the corresponding simulative materials were 0.33, 0.22, and 0.11 Mpa, considering a simulative stress ratio of 1:54.

Based on the results of the experiment in Table 1, bearing stress should be within 1 MPa. The proportion of materials of simulative siltstones, mudstones, and raw coals were 12:1:1.2; 14:1:1.4, 16:1:1.6 in order to satisfy the division of roadways bearing structure and to meet the need of observing the transformation and development of roadways. The respective compressive strengths were 0.404, 0.232, and 0.127 MPa, which all meet existing standards.

In this model, river sand was selected as the aggregate, and the cementing materials were lime and gypsum. According to the ratios summarized from long-term laboratory tests, the ratio type closest to the rock mass strength of this test was identified, and the ratio of similar materials was calculated based on this^27^, as shown in Table 3.

**Table 3:** Comparison between actual parameters and model parameters.

Parameter	Field parameters				Model parameters				
	Lithology	Thickness /m	Uniaxial compressive strength/ MPa	Unit weight/ 104 *N*/m3	Thickness /cm	Uniaxial compressive strength/MPa	Unit weight/ 104 *N*/m3	Mix ratio number	Water volume/Kg
Roof	Fine sandstone	9.00	22.00	2.54	9.00	0.13	1.54	1237	34.57
	Siltston	6.00	30.00	2.51	6.00	0.18	1.53	1237	7.92
	Fine sandstone	4.00	63.00	2.56	4.00	0.37	1.56	873	3.15
	Siltstone	10.00	49.60	2.57	10.00	0.29	1.56	1037	3.77
	Medium-fine sandstone	3.00	72.48	2.58	3.00	0.42	1.57	873	17.57
	Mudstone	5.00	28.41	2.45	5.00	0.17	1.49	1237	1.41
Coal seam	8coal seam	8.00	8.20	1.40	8.00	0.09	0.85	1237	3.86
Floor	Mudstone	4.00	30.30	2.49	4.00	0.18	1.51	1237	2.65
	9coal seam	4.00	30.30	2.49	4.00	0.18	1.51	1237	2.65
	Aluminous mudstone	3.00	31.60	2.50	3.00	0.19	1.52	1237	2.00
	Mudstone	5.00	30.30	2.49	5.00	0.18	1.51	1237	2.65
	Siltstone	5.00	49.60	2.57	5.00	0.29	1.56	1037	1.69
	Mudstone	5.00	12.30	2.61	5.00	0.07	1.59	1237	1.29
	Siltstone	7.00	49.60	2.57	7.00	0.29	1.56	1037	2.00
	Mudstone	8.00	12.30	2.61	8.00	0.07	1.59	1237	1.29
	Siltstone	4.00	47.68	2.62	4.00	0.27	1.59	1037	2.64
	Mudstone	5.00	28.40	2.55	5.00	0.17	1.55	1237	3.19
	Siltstone	2.00	47.68	2.62	2.00	0.27	1.59	1037	1.30
	Mudstone	5.00	12.30	2.61	5.00	0.07	1.59	1237	1.89
	Sandy mudstone	3.00	12.30	2.61	3.00	0.07	1.59	1237	1.89

### Boundary stress conditions and monitoring point layout of the test model

**Fig. 4:**
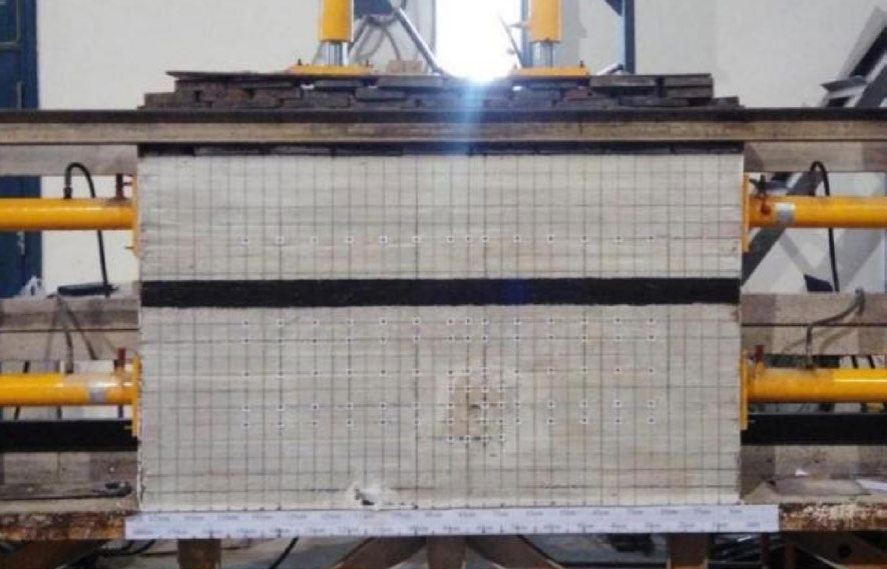
Plane stress model experiment rig.

The test is carried out using a plane stress test bench with lateral loading function. The test bench is equipped with two hydraulic jacks in the vertical direction to apply vertical stress, and two hydraulic jacks on each side of the horizontal direction to apply horizontal stress, as shown in Fig. 4. The geometric dimensions of the model are (length × width × height) 180 cm × 30 cm × 105 cm. According to the geometric similarity ratio of 1:100.

#### Boundary stress conditions

To simulate the self-weight stress of the overlying rock strata in the vertical direction of the prototype, according to the geological data of the target mining area, the burial depth of the prototype roadway is 500 m. The vertical stress of the prototype σ_v−p_ = γ_p_ × H = 25 KN/m^3^ × 500 m = 12.5Mpa, Combined with the stress similarity ratio of 1:54, the vertical boundary loading stress of the model σ_v−m_ = 12.5\ MPa/54 ≈ 230 kPa, This stress is applied synchronously by 2 jacks on the top plate of the test bench, and a stepwise pressure stabilization method is adopted during the loading process.

To simulate the horizontal horizontal tectonic stress of the prototype, with reference to the in-situ stress test report of the mining area, the horizontal principal horizontal stress of the prototype σ_h−p_ is 15 MPa (with a lateral pressure coefficient of 1.2). Correspondingly, the horizontal boundary loading stress of the model σ_h−m_ is calculated as approximately 278 kPa by dividing 15 MPa by the stress similarity ratio of 54. This stress is applied symmetrically by jacks on both sides of the model, and the loading rate is kept consistent with that in the vertical direction to ensure that the synergistic effect of boundary stresses conforms to the characteristics of the prototype in-situ stress field.

####  monitoring point layout of the test model

**Fig. 5:**
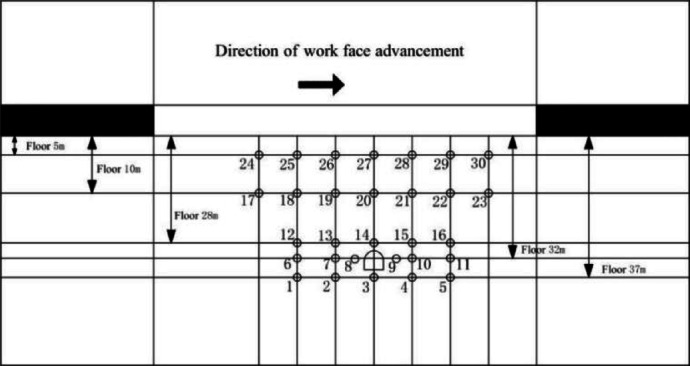
Schematic diagram of monitoring points layout.

In this simulation test, the stress in the coal seam floor was real-time tracked and monitored during the coal seam mining process. The stress monitoring of this test group was completed using BX120-50AA type resistance strain gauges and CM-2B-64 program-controlled static resistance strain meters. The layout of stress measurement points is shown in Fig. 5. Five measuring lines (30 points in total) are arranged in the horizontal direction, at distances of 37 cm, 32 cm, 28 cm, 15 cm, and 5 cm from the floor of Coal Seam 8 respectively. The measuring points are numbered in natural order from left to right along each measuring line. For example, the points on the first line are labeled as 1, 2, …, 5 (5 points in total), and the same principle applies to the second and third lines.

### Test results and analysis

#### Model observation during coal seam mining process

**Fig. 6:**
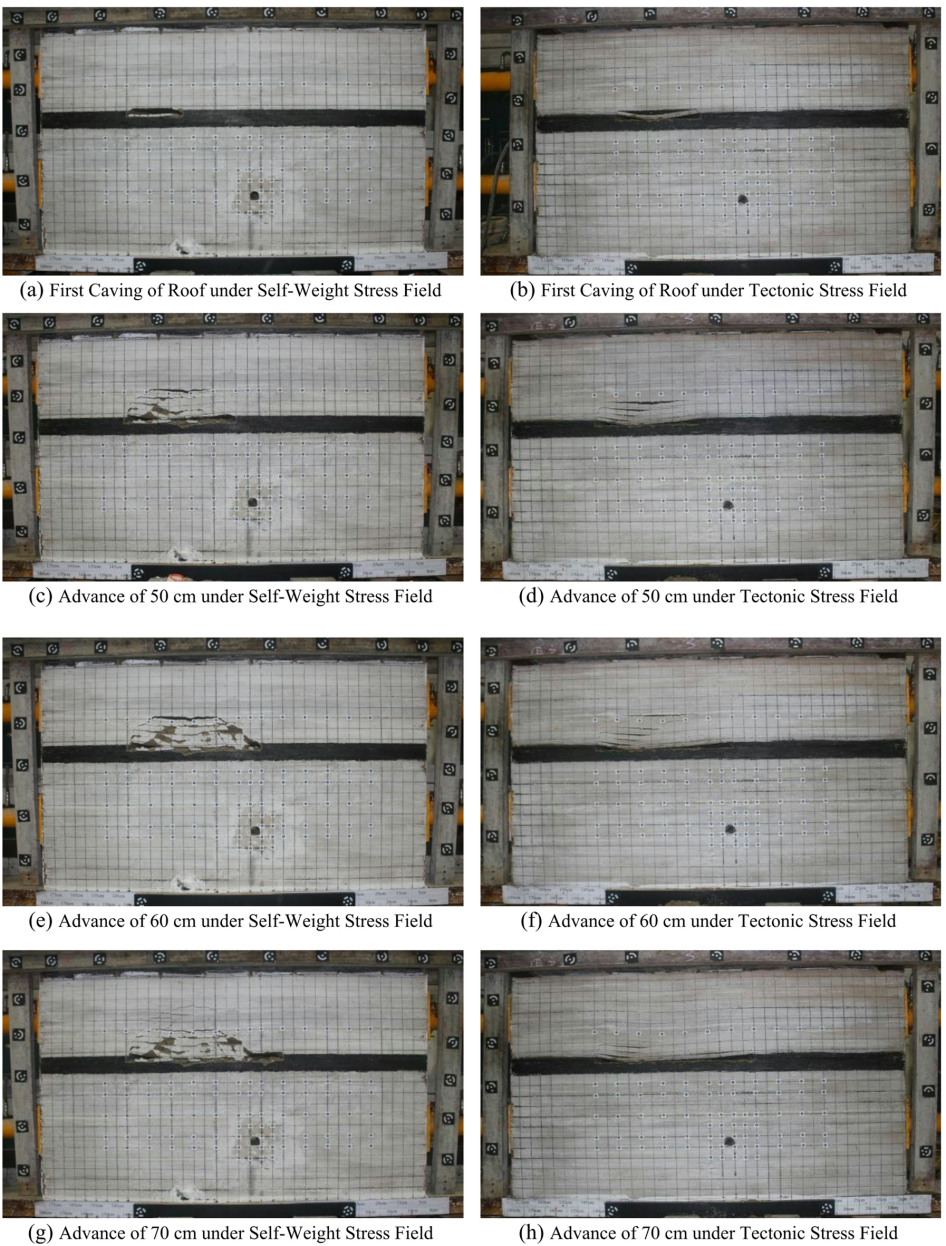

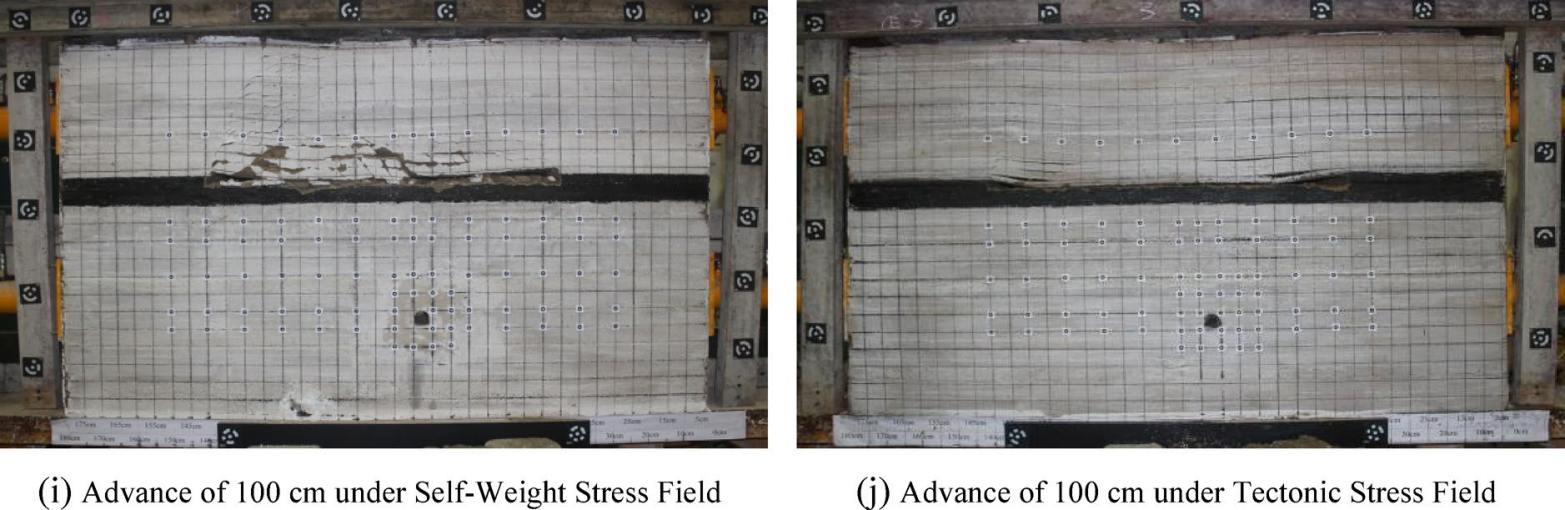
Deformation feature of the model during coal mining.

In this model, the coal seam is relatively thick at 8 cm, and only the upper layer is mined during the mining process, with a mining thickness of 3 cm. A comparative study was conducted on the changes in the model during coal seam mining under different stress field conditions. Figures 6a-b show the first caving conditions of the coal seam roof under different stress conditions. It can be seen that the first caving step distances of the two models differ, in the model without lateral pressure, the roof first caves when the coal seam is mined 25 cm, while in the model with lateral pressure, the roof caves only when the coal seam is mined to 40 cm. This indicates that the horizontal squeezing effect of the tectonic stress field increases the first caving step distance of the roof. Figures 6c-d show the conditions when the working face is advanced by 50 cm. Through comparison, it is found that under the same advancing step distance, in-situ stress has a significant influence on the roof caving height. Under the self-weight stress field condition, the height of the caving zone is 14 cm, while under the tectonic stress field condition, the height of the caving zone is only 9 cm. When the working face advanced from 60 cm to 70 cm, under the self-weight stress field condition, the roof delamination phenomenon continued to develop, while under the tectonic stress condition, the roof delamination began to close under the lateral squeezing effect, as shown in Figs. 6e–h.The morphology of the model after mining is shown in Figs. 6i–j. The morphological characteristics of roof failure under the two stress conditions are significantly different.

#### Evolution characteristics of mining-induced stress in coal seam floor

**Fig. 7:**
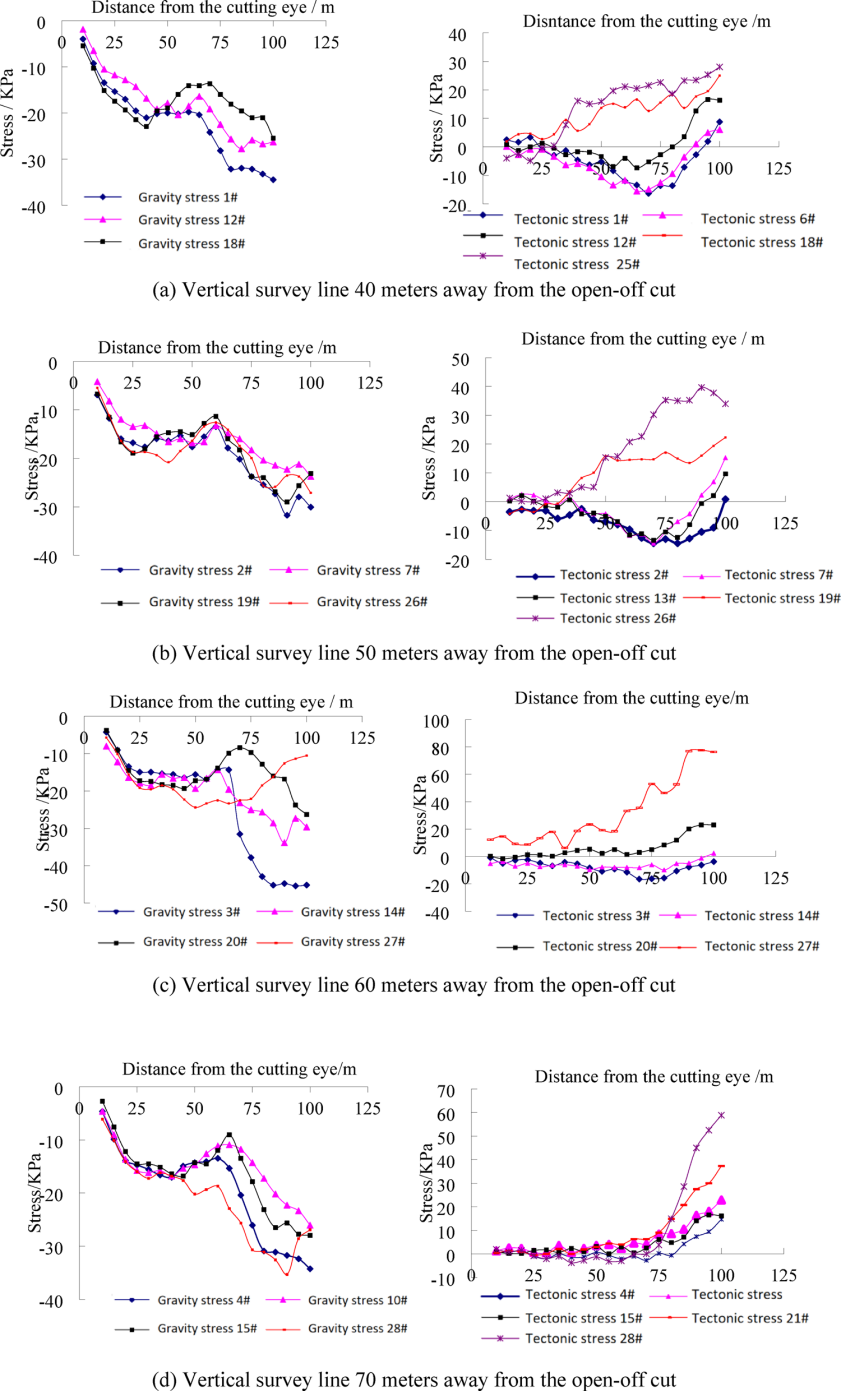
The stress evolution law of floor strata under different stress field during coal mining.

To study thehe monitoring results of stress measurement points in the floor were analyzed. The measurement points in the vertical direction were selected as the survey line for analysis. As a very small number of strain gauges were damaged, this study only focuses on valid monitoring data. The specific results are shown in Fig. 7.

It can be seen from the mining-induced stress curve of the coal seam floor that the in-situ stress field has a significant controlling effect on the evolution of mining-induced stress. Under the condition of self-weight stress field, the variation laws of survey lines at different distances from the open-off cut are basically consistent. The overall stress variation law below the coal seam floor can be divided into four stages: pressure relief - stress. The specific manifestations are as follows: The first stage of pressure relief is caused by the pressure relief of the goaf formed after coal seam mining, and the farther away from the goaf, the smaller the degree of pressure relief influence. After that, the roof collapses and is gradually compacted, and the stress gradually stabilizes. When the working face advances 60 m, obvious pressure increase occurs in the stress at different depths of the floor. Comparing with the model excavation diagram 6-e, it can be seen that at this time, a large-scale roof collapse occurs, and a stress fulcrum is formed near 65 m from the open-off cut. Under the combined influence of the roof impact stress and the supporting effect, the stress increases. Finally, with the advancement of the working face, obvious suspended roof is formed, causing pressure relief in the goaf, and then causing pressure relief in the entire floor.In addition, there are differences in the variation laws at different depths of the floor, indicating that the mining-induced stress changes with depth. Through comparison, it can be seen that the pressure increase range of the 18#–20# measuring points below the floor is the largest. These measuring points are located 15 m below the coal seam floor, while the pressure relief degree of the measuring points 5 m below the floor is the highest. This shows that during the coal seam mining process, the area 15 m below the floor is most significantly affected by pressure increase. Under the condition of tectonic stress field, the stress variation of the measuring points on the same survey line is obviously different from that under the condition of self-weight stress field. The variation laws of the three vertical survey lines on the left side of the roadway are basically consistent. As can be seen from Fig. 7a and b, under tectonic stress conditions, there are obvious stress differences below the floor, mainly manifested in that the measuring points No. 18, 25, 19, 26 and 20, 27 are in a pressure-increasing state. That is, affected by tectonic stress, the stress state of the coal seam floor above 15 m is compressive. In contrast to the self-weight stress condition, the floor stress is in a tensile state, indicating that the pressure relief phenomenon of the goaf floor is not obvious under the action of lateral tectonic stress. Below 15 m of the floor, the stress state of each measuring point is tensile, but the degree of tension is smaller compared with the self-weight stress field. Under the action of tectonic stress, 15 m below the floor is an obvious stress conversion zone, which is the pressure-tension boundary of the floor.

#### Evolution law of mining-induced stress in surrounding rock of floor roadway

**Fig. 8:**
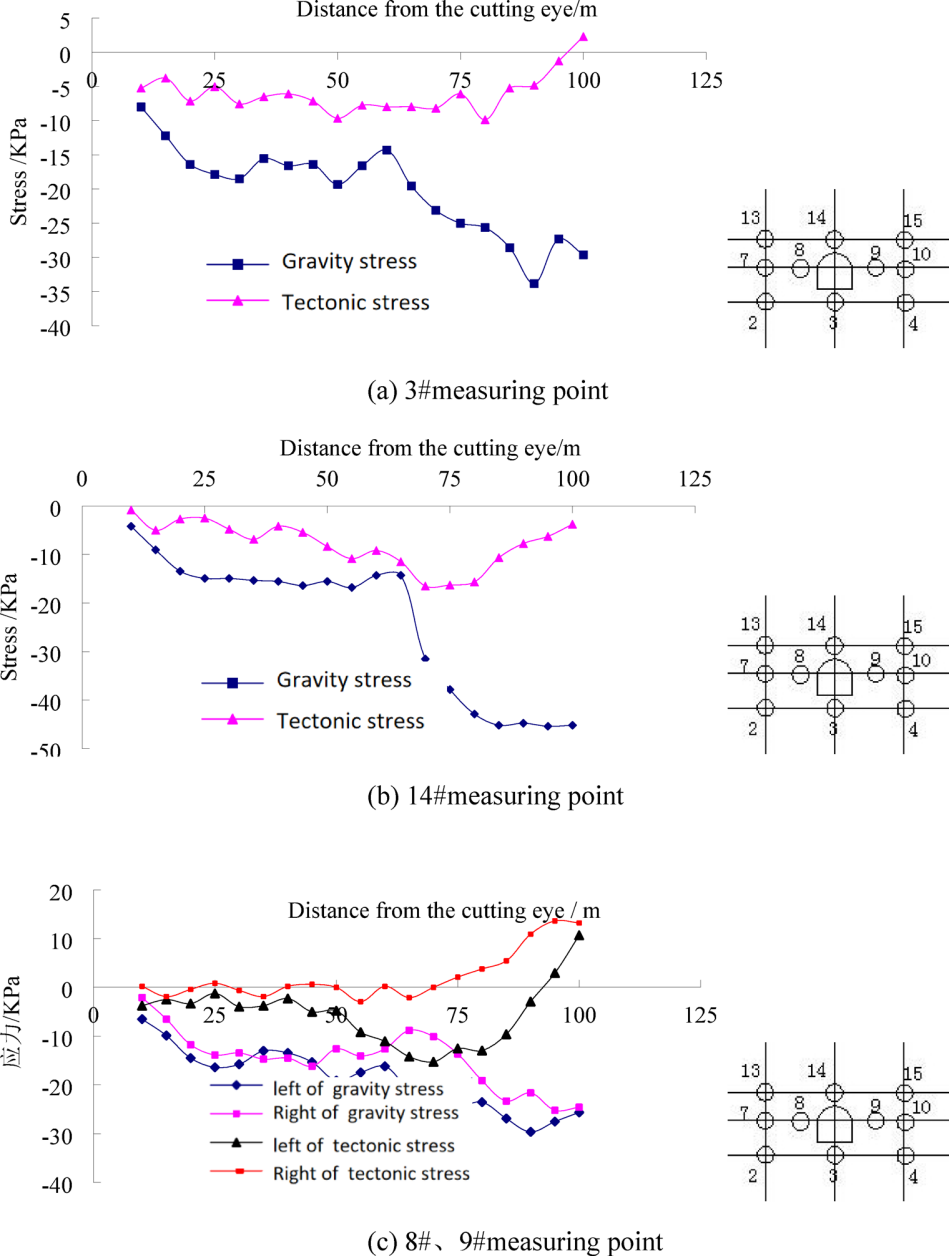
Stress variation characteristics of roadway surrounding rock under different stress field.

According to the arrangement of stress measuring points in the model, the monitoring results of the measuring points on the roof, floor, left side and right side of the roadway are analyzed with emphasis, so as to compare the influence of working face mining on the stress transfer of surrounding rock in the floor roadway under different in-situ stress field conditions. The results are shown in Fig. 8.

From the perspective of the influence of working face mining on floor roadways under different in-situ stress field conditions, in-situ stress has an obvious controlling effect on roadway deformation, which is mainly manifested in that under the same mining conditions, different in-situ stresses lead to significant differences in mining-induced stress of roadway surrounding rock.

1. Stress at the roadway bottom. Under the condition of self-weight stress field, the stress variation in the roadway floor is minimal in the early stage. With the advancement of mining in the overlying coal seam, obvious pressure relief occurs in the floor stress, and the degree of pressure relief is far greater than that under tectonic stress conditions. After that, the stress basically remains unchanged without obvious stress recovery. Under the action of tectonic stress, with the advancement of mining in the overlying coal seam, when the roadway is located below the goaf, it is affected by floor pressure relief, and the stress drops to the minimum at this time. Afterwards, the stress gradually recovers due to the compaction effect caused by the periodic collapse of the roof.
2. For the roadway roof, the monitoring results show that: with the advancement of the working face, under the tectonic stress field, the roof stress changes little. After the working face advances 80 m, there is an obvious pressure increase. Under the self-weight stress condition, the initial stress change is also small. When the working face advances 50 m, that is, when the measuring point is 10 m in front of the working face, the pressure increase begins, mainly due to the influence of the advanced concentrated stress of the working face. When the working face advances 60 m, that is, when the measuring point is located in the goaf of the working face, affected by the floor pressure relief, the roof of the roadway also shows pressure relief. The existence of the tectonic stress field weakens the influence of the advanced stress of the working face.
3. For the left and right sides of the roadway, the monitoring results show that: Under the self-weight stress condition, the stress variation laws of the left and right sides are basically consistent, but the right side is more obviously affected by the advanced concentrated stress of the working face, with a larger pressure increase range than the left side. This is mainly because the measuring points on the right side are farther from the working face heading, leading to higher stress concentration. Under the tectonic stress field condition, the stress variation laws of the left and right sides differ significantly. Specifically, the stress on the left side undergoes a pressure relief stage due to the pressure relief effect of the goaf, followed by a stress increase and recovery, while the right side does not exhibit obvious pressure relief. For the same side under different stress conditions, the stress variation laws also differ significantly. Notably, after the working face passes the roadway, obvious pressure increase occurs on both sides under tectonic stress conditions, whereas obvious pressure relief occurs under self-weight stress conditions.

Therefore, due to differences in stress states and magnitudes of stress changes, the deformation and failure modes of the roadway may vary. Through analysis, under the action of a tectonic stress field, the roadway is likely to undergo deformation and failure dominated by compressive-shear failure, while under self-weight stress conditions, tensile cracks may occur in the two sidewalls of the roadway. The smallest stress fluctuation in the roadway roof indicates the lowest degree of deformation and failure in the roof. The stress variation range in the roadway floor is moderate, and its degree of deformation and failure also lies between the two extremes.

## Numerical simulation study

### Technical background of numerical model setup

**Fig. 9:**
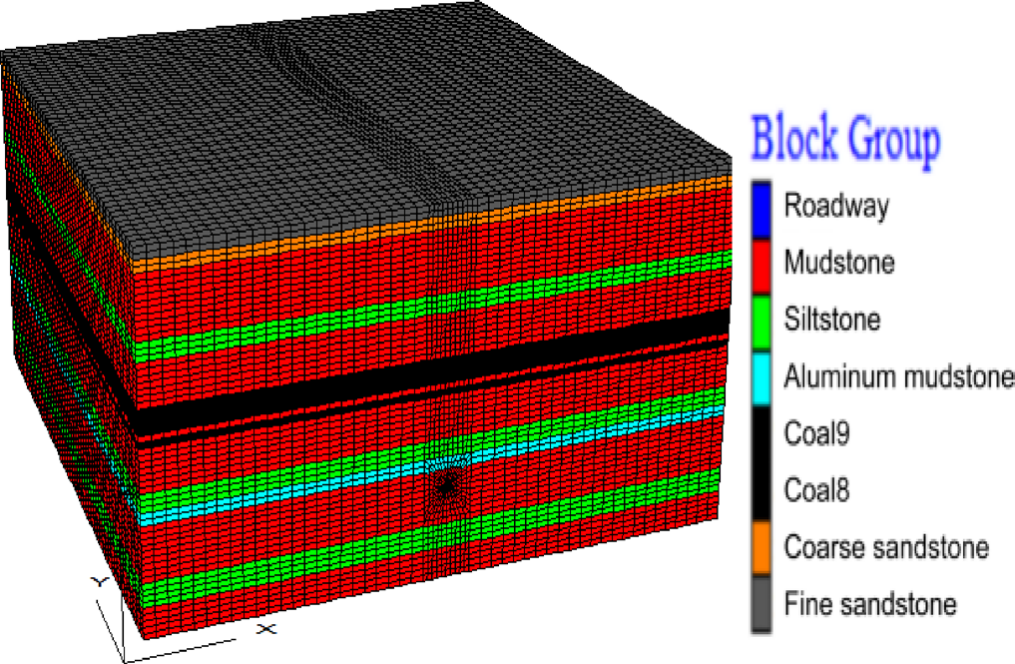
Numerical model of full mudstone stratum structure floor.

According to the actual mining conditions of the working face in Luling Coal Mine and the engineering geological model of the soft rock formation floor, the numerical model is set with X, Y, and Z dimensions of 250 m, 250 m, and 120 m respectively. The floor roadway is located approximately 35 m below the No. 8 coal seam, with an arched roadway section. The roadway dimensions are: 3.5 m in height and 4.0 m in width, with local grid encryption performed near the floor roadway. The average mining thickness of the coal seam is 3 m, the burial depth is 500 m, and the simulated formation dip angle is 0°.The model is composed of 178,500 cells and 187,527 unit nodes, and the numerical model is shown in Fig. 9.

The mechanical parameters of the rock mass adopted in the numerical simulation are shown in Table 4.

**Table 4:** Mechanical param of numerical model.

Lithology	Bulk Modulus /GPa	Shear Modulus /GPa	Density /g·cm^− 3^	Cohesion /MPa	Internal Friction Angle /°	Tensile Strength /MPa
Coal 8	0.09	0.08	1.38	1.25	32	0.25
Coal 9	0.09	0.08	1.38	1.25	32	0.25
Mudstone	1.19	1.26	2.38	2.00	30	1.50
Roadway	1.19	1.26	2.38	2.00	30	1.50
Aluminous Mudstone	1.29	1.36	2.48	2.10	31	1.60
Siltstone	2.58	1.59	2.46	2.80	33	2.30
Fine Sandstone	1.54	1.88	2.54	3.00	35	2.40
Coarse Sandstone	3.61	2.5	2.89	1.98	35	3.20

**Fig. 10:**
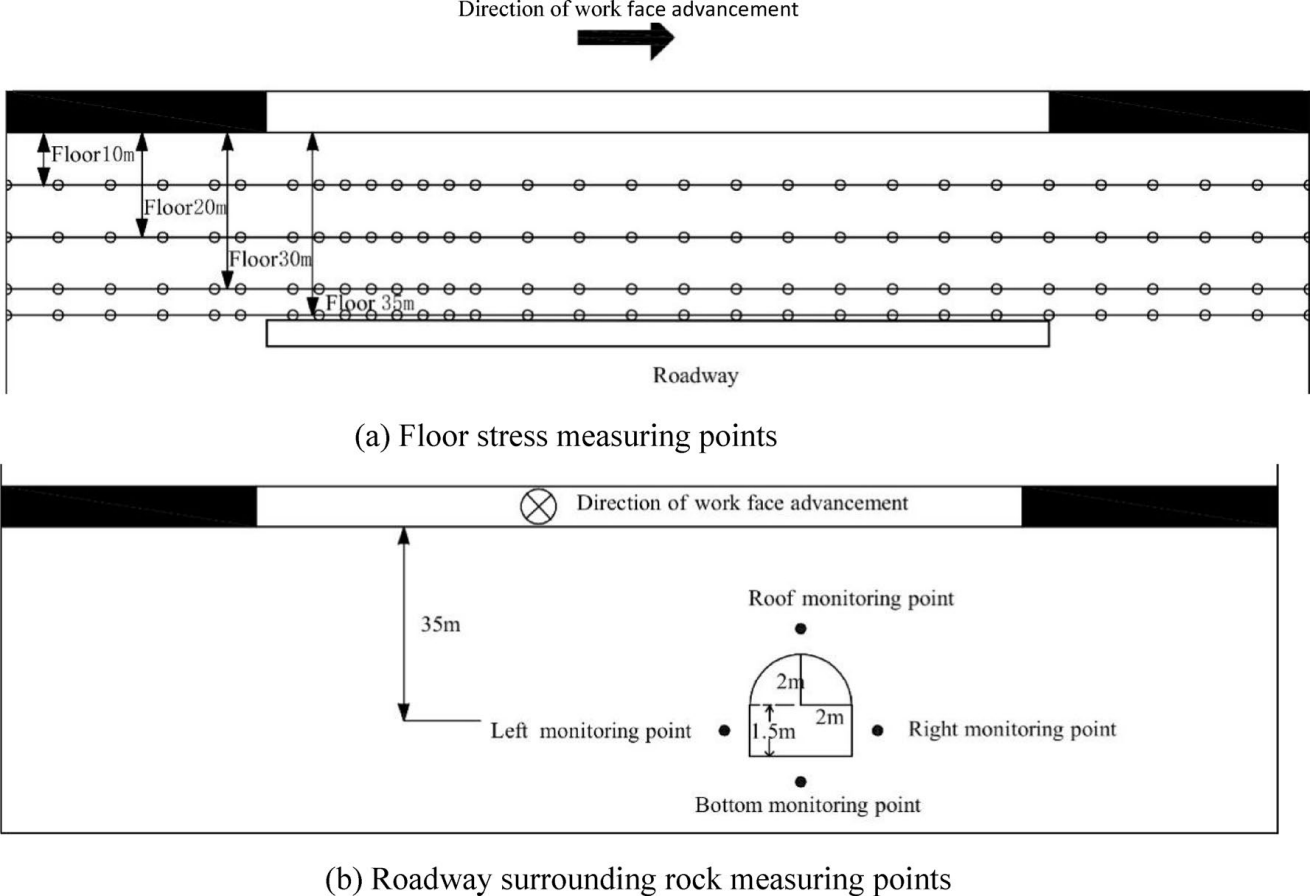
Schematic diagram of monitoring point layout for coal seam floor.

The model boundary adopts full constraints at the bottom, free boundary conditions at the top, and fixed constraints in the X and Y directions for the front, rear, left, and right sides. The model employs the Mohr-Coulomb plastic constitutive model; the model yield criterion adopts the Mohr-Coulomb yield criterion. The working face mining is carried out by step-by-step excavation. According to the roof caving law during the mining of the coal seam working face in the mine, the initial weighting of 30 m and the periodic weighting of 20 m are adopted for excavation. The roof is subjected to the free caving method, and the working face width is 150 m. To eliminate the boundary effect, constrained coal pillars of 50 m are reserved on the front, rear, left and right sides of the excavation space. The mining direction of the working face is consistent with the axial direction of the roadway. In the model, the axial direction of the roadway is along the Y-axis of the model, and the mining direction of the working face is also in the Y-axis direction. Schematic diagrams of stress measuring points at different depths of the floor and displacement measuring points of the roadway surrounding rock are shown in Fig. 10.

### Analysis of numerical simulation results

Before the coal seam is mined, the surrounding rock stress is in a relatively balanced state. After the coal seam is mined, the stress of the rock mass around the goaf is redistributed. The floor stress below the goaf is released, forming a pressure relief zone; The rock mass above the goaf forms a load-bearing rock stratum, which is transferred to the surrounding rock around the goaf, forming a stress concentration area higher than the original rock stress, and distributed in a certain law, that is, stress concentration is formed within a certain range around the goaf, and pressure relief is formed within a certain range of the floor, both of which weaken with the increase of distance. The floor stress is constantly adjusted and changed with the advancing step of the working face. The stress adjustment and distribution are the driving forces leading to the plastic failure and deformation displacement of the roadway surrounding rock.

#### Analysis of stress evolution and plastic failure in the stope floor under self-weight stress field conditions

**Fig. 11:**
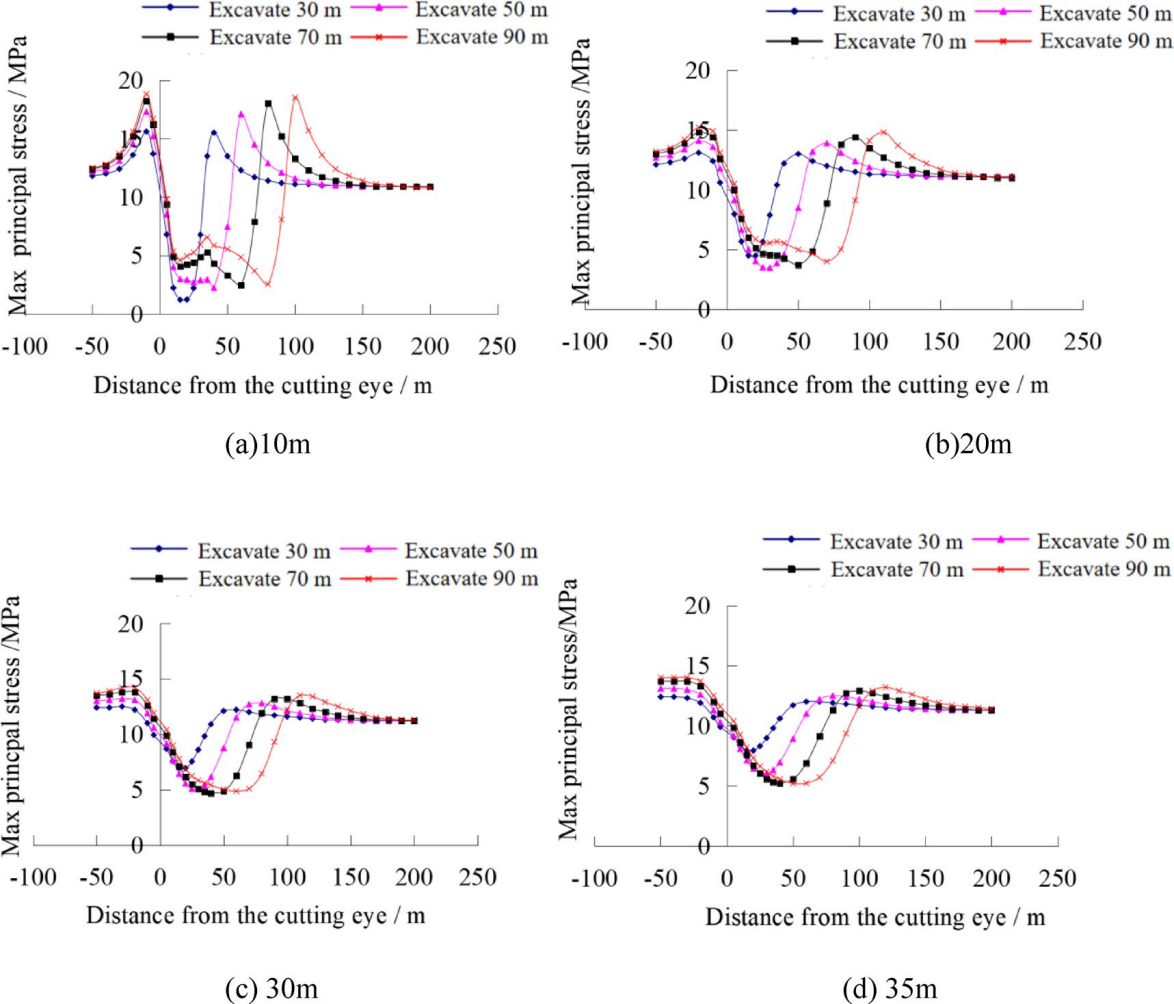
Evolution law of maximum principal stress at different depths of the floor.

By extracting the maximum principal stress values from stress monitoring points at different depths, the variation curves of the maximum principal stress at different depths of the coal seam floor after mining can be obtained, as shown in Fig. 11. Divide the floor in front of the working face into four parts horizontally: ①Advanced yield zone, within 0–8 m in front of the working face, the coal mass at the edge of the working face is crushed and undergoes yield failure, reducing its ability to bear the load of the overlying strata; ②Advanced abutment pressure zone, in the range of 8–38 m in front of the working face, affected by the mining disturbance, the stress increases rapidly and reaches the peak value of the abutment pressure; ③Stress micro-variation zone, within the range of 38–78 m in front of the working face, the stress of the floor slightly increases due to the mining influence; ④Original rock stress zone, beyond 78 m in front of the working face, the floor is basically unaffected by mining-induced stress and remains in the original rock stress state. As the working face advances, the stress peak also moves forward continuously, with a slight increase in the stress amplitude. The vertical stress curve is basically symmetrically distributed around the goaf.

As shown in Fig. 11, the greater the depth from the coal seam floor, the smaller the amplitude of stress change in the floor rock mass, and the pressure relief amplitude gradually weakens. The advanced stress concentration coefficients of the floor at 10 m, 20 m, 30 m, and 35 m from the working face are 1.42, 1.17, 1.08, and 1.06, respectively. That is, the farther away from the working face, the smaller the influence of mining on the floor.

**Fig. 12:**
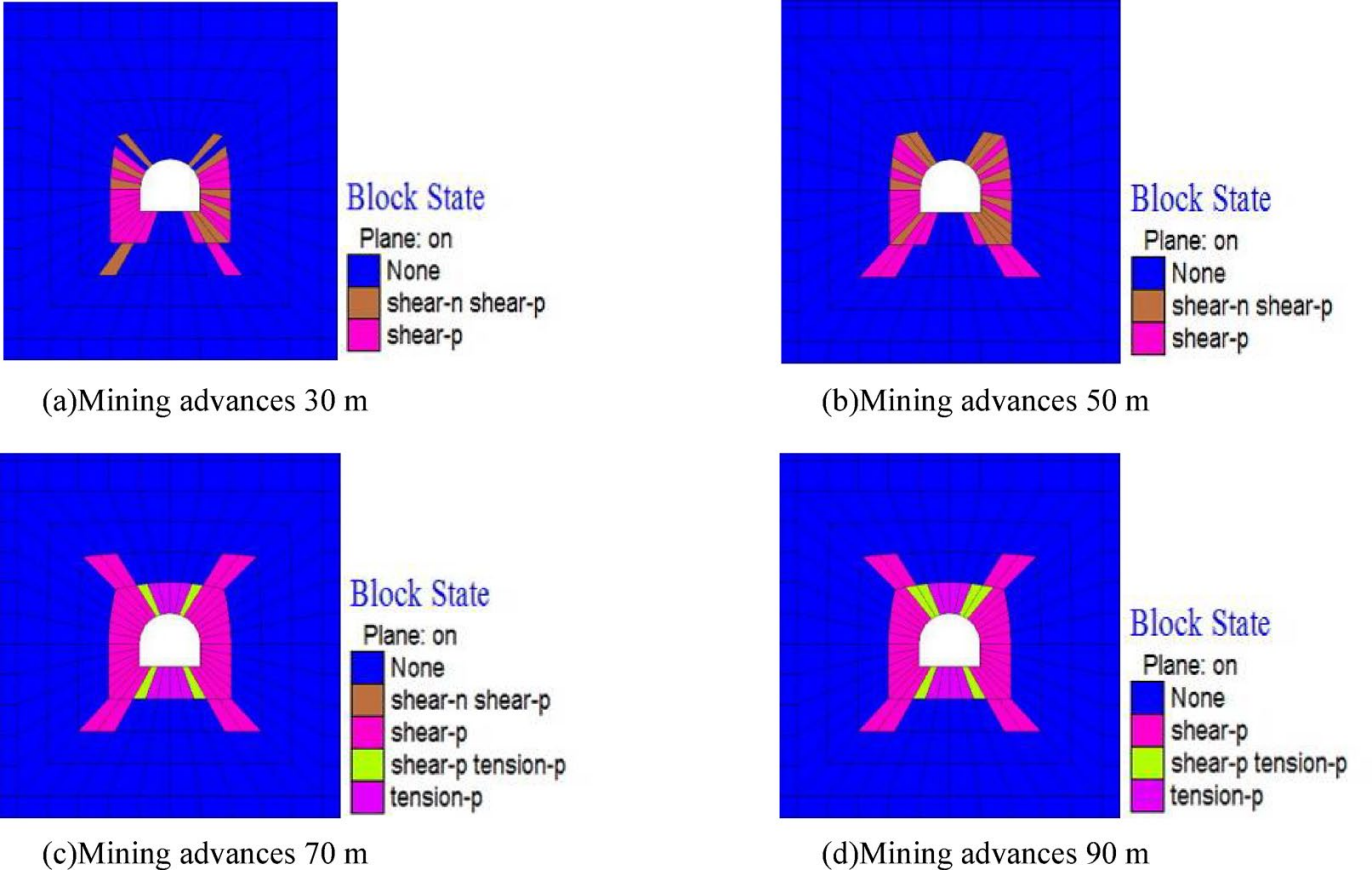
Plastic zone nephogram of surrounding rock of floor roadway.

Figure 12 shows the nephogram of plastic zone changes in the roadway analysis section with the mining of the working face. It can be seen that: When the working face advances 30 m, the shear failure of the roadway surrounding rock increases; When advancing 70 m, the shear failure of the roadway surrounding rock further intensifies, and tensile failures occur at the bottom corners and arch shoulders of the roadway; With the further advancement of the working face, the plastic failure zone of the roadway surrounding rock remains unchanged.

By comparing the plastic failure of the roadway surrounding rock before and after the mining of the working face, it can be seen that the scope of the plastic failure zone in the surrounding rock of the floor roadway significantly increases under the influence of the working face mining.

#### Analysis of stress evolution and plastic failure in the stope floor under tectonic stress field conditions

**Fig. 13:**
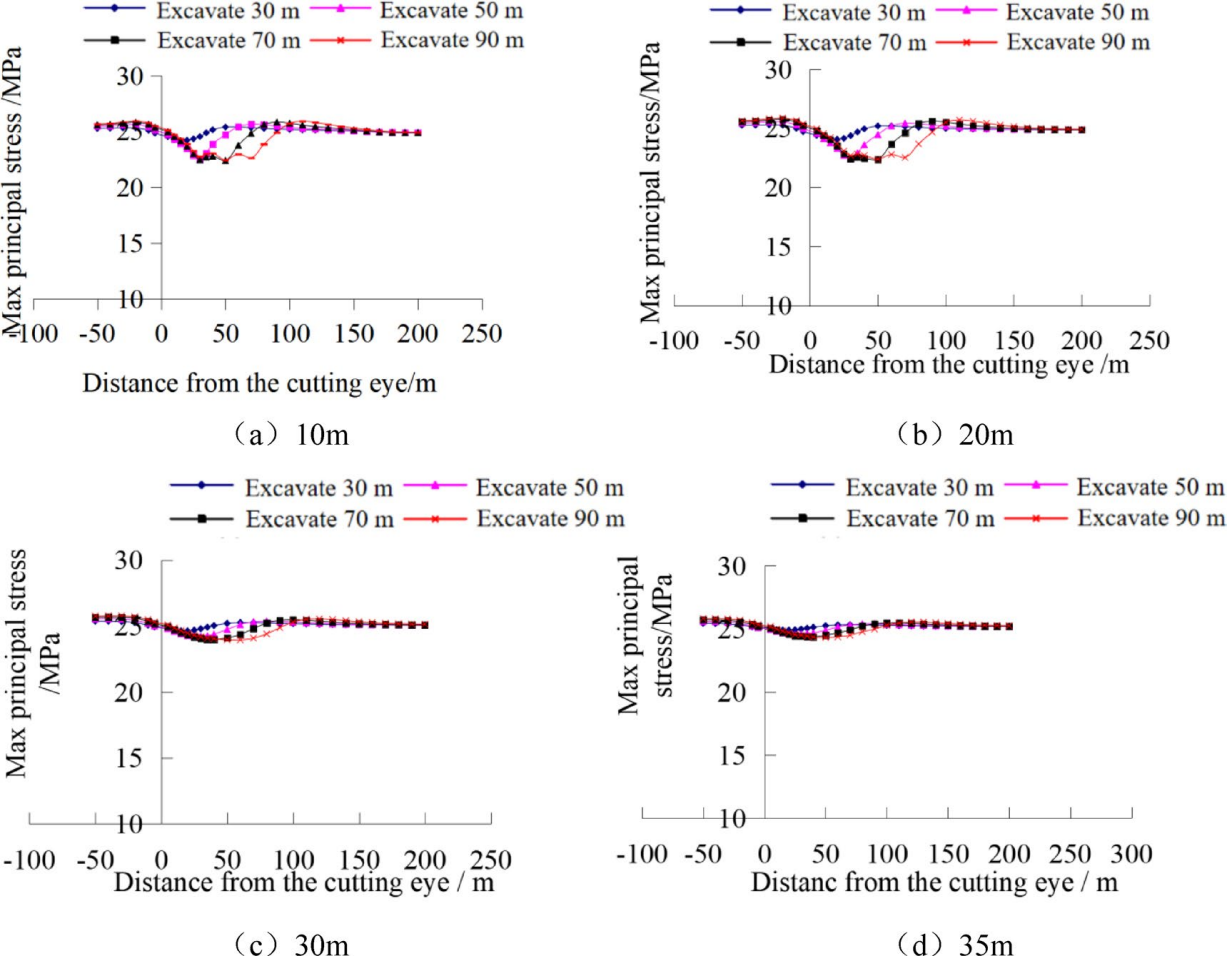
Evolution law of maximum principal stress at different depths of the floor.

Under tectonic stress field conditions, the maximum principal stress values were extracted from stress monitoring points at different depths of the floor, as shown in Fig. 13. It can be seen that with the increase of floor depth, significant differences appear in the stress changes of the stope, mainly manifested as the farther away from the coal seam, the lower the degree of influence by mining, and both the advanced concentrated stress in front of the working face and the pressure relief degree of the floor decrease. Below 30 m of the floor, the stress curve is basically a horizontal straight line, revealing that the tectonic stress field “weakens” the degree of advanced stress concentration and floor pressure relief.

By comparing with Fig. 11 under the self-weight stress field, it can be seen that different in-situ stress fields lead to significantly different laws of floor stress evolution after working face mining, revealing that the in-situ stress field has an obvious controlling effect on the evolution of mining-induced stress in the floor.

**Fig. 14:**
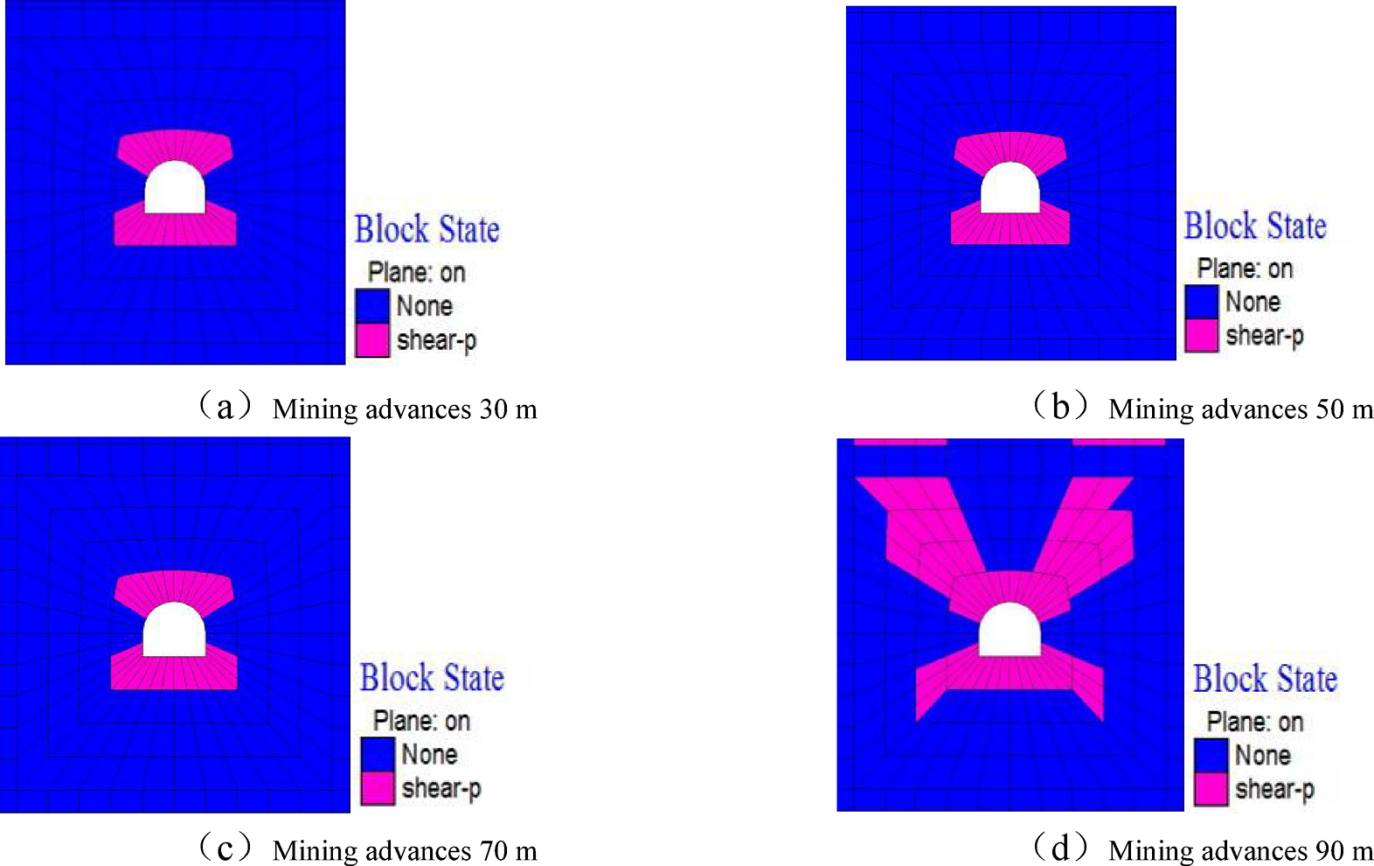
Plastic zone nephogram of surrounding rock of floor roadway.

Figure 14 shows the plastic change process of the roadway surrounding rock as the overlying working face advances. After the working face has advanced 70 m from the open-off cut, the roadway at the working face heading has maintained shear failure within a 2-meter range of the roof and floor. When the working face advances to 90 m, due to the pressure relief effect of the goaf, the plastic failure of the roadway changes significantly: plastic failure occurs within an 8-meter range of the roadway roof, especially at the arch shoulders of the roadway, where the damage is severe; within a 6-meter range of the roadway floor, the damage is particularly serious at the floor corners. As the working face continues to advance, the plastic failure zone basically stabilizes, mainly because the roadway gradually moves away from the working face and enters the stress recovery zone.

Under tectonic stress conditions, when the working face advances 90 m, the pressure-relief range of the goaf in the working face only affects the floor roadway, leading to a significant increase in the scope of the roadway plastic failure zone. Under self-weight stress conditions, when the floor advances 70 m, the pressure-relief zone of the goaf in the working face affects the floor roadway, causing an increase in the scope of its plastic failure zone. Comparing Fig. 11, before the working face advances 70 m (i.e., when the roadway is located at the working face heading), the plastic failure of the roadway in the self-weight stress field is mainly concentrated at the four arch corners, while in the tectonic stress field, the plastic failure is mainly concentrated in the roof and floor. In addition, after being affected by the unloading of the working face goaf, the change in the plastic range of the roadway under self-weight stress is not obvious, while the plastic zone in the tectonic stress field expands significantly, and the failure mode is mainly shear failure. In the self-weight stress field, the failure is mainly shear-tensile failure. Through analysis, it can be seen that different stress field environments lead to significant differences in the failure modes and scopes of stope floor roadways, revealing the control mechanism of the in-situ stress field on the failure of floor roadways.

## Conclusions

Through similar material model tests, numerical simulations, and studies on the stress evolution and plastic failure of roadway surrounding rock under stepwise mining and different in-situ stress controls, this paper draws the following conclusions:

1. The deformation and failure of stope floor roadways are affected by in-situ stress conditions: Under self-weight stress, stress concentration and plastic failure are more significant in the two sidewalls of the roadway, while the roof and floor exhibit less pressure relief and plastic failure; Under tectonic stress, stress concentration and plastic failure are less significant in the roof and floor, while the two sidewalls show more pressure relief and plastic failure.
2. The influence of working face mining on the surrounding rock of floor roadways is mainly manifested as: The degree of pressure relief effect is significantly greater than that of stress concentration, and this difference is amplified under tectonic stress. Affected by floor pressure relief, pressure relief also occurs in the roadway roof.
3. Under the same mining conditions, the stress concentration of the surrounding rocks under tectonic stress field was higher than that without tectonic stress. This phenomenon was more evident on the sides of roadway and at the end of the arch foot. However, the stress transferred more obviously and was prone to shear failure.
4. In the tectonic stress field, tensile stress is not obvious and compressive stress is dominant, so roadway failure is mainly compression-shear failure. In the self-weight stress field, tensile stress is dominant, so roadway failure is mainly tensile failure.
5. Through comparative analysis, this study investigates the roadway stress field and the deformation and failure laws of surrounding rock under the influence of different stresses, and reveals the control mechanism of overlying coal seam mining on the stability of floor roadways.

The similar simulation test adopted in this paper restored the soft rock stratum structure in the field. The selected similar materials were only matched in terms of main components and could not be completely consistent with those in the field. Therefore, there are slight differences in the magnitude of surrounding rock stress, but the change mechanism remains consistent. The concept proposed in this paper can provide a theoretical reference for roadway support under similar engineering geological conditions.

## Data Availability

The data used to support the findings of this study are avail able from the corresponding author upon request.
